# Downstream Processing of *Chlamydomonas reinhardtii* TN72 for Recombinant Protein Recovery

**DOI:** 10.3389/fbioe.2019.00383

**Published:** 2019-12-06

**Authors:** Laura Stoffels, Arran Finlan, Gareth Mannall, Saul Purton, Brenda Parker

**Affiliations:** ^1^Department of Biochemical Engineering, Bernard Katz Building, University College London, London, United Kingdom; ^2^Algal Research Group, Institute of Structural and Molecular Biology, University College London, London, United Kingdom

**Keywords:** algae, downstream processing, scale-up, ultra scale-down, disc-stack centrifugation, endolysin

## Abstract

The green microalga *Chlamydomonas reinhardtii* is under development as a production host for recombinant proteins and whole-cell therapeutics. In particular, the cell wall-reduced strain TN72 is used as a model organism for protein expression and algal synthetic biology. However, the bioprocessing characteristics of TN72 and other *C. reinhardtii* strains have yet to be examined. Here we use a TN72 strain expressing a protein-based antibiotic (Pal) to study the scale-up of cell harvest and product recovery. Cell harvest was examined with 100L cultures in two intermittent-discharge continuous-flow disc-stack centrifuges at flow rates of 150–250 L.h^−1^, as well as with an ultra scale-down (USD) mimic of the centrifuges. Solids recovery exceeded 99.5% and the loss of product to the supernatant was below 2–3%. TN72 is intact following the high shear conditions of the feed zone, however discharge from both disc-stack centrifuges resulted in full cell breakage and in the case of Pal, partial degradation in the subsequent hours. We demonstrated that shake flask cultivation and the USD centrifuge technique can be used to predict the pilot-scale clarification efficiency and product release at the centrifuge inlet for TN72, but not the cell breakage on discharge. This study outlines a number of challenges for scale-up of recombinant protein production in the microalgal host in particular for whole cell therapeutics, but also opportunities for the bioprocessing of intracellular products from TN72.

## Introduction

Microalgae represent an emerging biotechnology platform for the light-driven synthesis of therapeutic proteins such as vaccines, antibodies and antimicrobials (Hempel and Maier, 2016; Taunt et al., 2017). Microalgae hold several advantages over other eukaryotic hosts for the production of recombinant proteins. These include simple and inexpensive nutrient requirements, which are believed to result in lower operating costs for upstream processing, and potentially in cost-efficient recombinant protein production (Gimpel et al., 2015). Scalable production systems for a number of microalgae exist (Borowitzka and Vonshak, 2017).

Compared to transgenic plants, microalgae can be easily grown in full containment under sterile conditions in simple photobioreactors (PBRs) (Specht et al., 2010; Rasala and Mayfield, 2015), which prevents the release of transgenes to the environment. In contrast, a major concern regarding the open and semi-enclosed cultivation of transgenic plants in greenhouses is the potential flow of transgenes to surrounding plants including food crops (Dove, 2002). Furthermore, growth in PBRs reduces the risk of environmental contamination of the production system and therefore reduces the risk of contamination of the therapeutic protein. Whereas the open and semi-enclosed cultivation of transgenic plants carries a high risk of infections with parasites and pathogens, which can result in the loss of the production crop (Specht et al., 2010).

Several species of microalgae are classified as GRAS (Generally Recognized as Safe) organisms, which means these organisms are safe for human consumption. Furthermore, these microalgae are free of endotoxins and viral or prion contaminants, which is important especially for the production of therapeutic proteins. The green microalga *Chlamydomonas reinhardtii is* a GRAS organism and toxicological studies have demonstrated that it is suitable for nutritional use (Murbach et al., 2018). Moreover, *C. reinhardtii* offers already established genetic tools including routine methods for transgene expression in the chloroplast (Mussgnug, 2015; Scaife et al., 2015; Taunt et al., 2017). Engineering of the chloroplast genome is particularly attractive as transgenes can be targeted to specific loci within the polyploid genome, and high levels of expression achieved without issues of instability since the chloroplast lacks the gene silencing mechanisms found in the nucleus (Bock, 2015). Whilst microalgal platforms such as *C. reinhardtii* have yet to be commercialized, there have been a growing number of reports describing the expression of recombinant proteins in the algal chloroplast, including numerous therapeutic proteins (Dyo and Purton, 2018). These include monoclonal antibodies (Tran et al., 2009), fusion-protein vaccines (Gregory et al., 2013), immunotoxins (Tran et al., 2013) and P450 enzymes to produce bioactive terpenoids (Gangl et al., 2015).

In order to simplify the delivery of foreign DNA into the chloroplast in *C. reinhardtii*, cell wall-reduced strains such as cw15 have been exploited that lack major parts of the multi-layered proteinaceous cell wall (Davies and Plaskitt, 1971; Hyams and Davies, 1972). Furthermore, such strains have been further developed as recipients for chloroplast engineering by deleting a chloroplast gene such as *psbH* that is essential for phototrophic growth. Transformants can therefore be selected based on restoration of phototrophy using the wild type *psbH* as the selectable marker (Economou et al., 2014). This strategy allows the generation of transgenic lines that are devoid of any undesirable markers such as those based on bacterial antibiotic resistance genes (Esland et al., 2018). One such cw15, Δ*psbH* recipient strain is TN72, which has been used for production of various proteins (Gangl et al., 2015; Zedler et al., 2015; Wannathong et al., 2016)

Recently, the TN72 strain has been used as an expression host for the production of bacteriolytic enzymes derived from bacteriophage viruses (Stoffels et al., 2017). There is a pressing need for the development of novel classes of antibacterials to combat growing antibiotic resistance amongst pathogens (World Health Organisation, 2014). Endolysins represent a promising class of protein-based antibiotics owing to their species specificity and demonstrated efficacy both *in vitro* and *in vivo*. Endolysins are bacteriophage-encoded enzymes produced at the end of the lytic cycle that facilitate the release of the progeny by catalyzing the breakdown of the peptidoglycan layer of the bacterial cell wall. Most endolysins have a two-domain structure with a N-terminal catalytic domain and a C-terminal peptidoglycan-binding domain (Fischetti, 2005; Borysowski et al., 2006). Exogenous application of endolysins to Gram-positive pathogens such as *Streptococcus pneumoniae* or *Staphylococcus aureus*, still enables rapid lysis as Gram-positive species lack a protective outer membrane. Previous attempts at recombinant expression of endolysins have been mainly done intracellularly in *Escherichia coli* (Haddad Kashani et al., 2018). Most recently, this has been carried out for the endolysins AP50-31 and LysB4 against *Bacillus anthracis* (Park et al., 2018). Furthermore, the recombinant expression of endolysin LysH5 against Staphylococcal species in lactic acid bacteria has been attempted previously. However, it has proved challenging to produce the protein in a soluble form and to achieve secretion in lactic acid bacteria (Rodríguez-Rubio et al., 2012). In our previous study we created transgenic lines of TN72 producing the *S. pneumoniae*-specific endolysins Pal and Cpl-1, quantified the production as ~1 mg endolysin/g biomass and demonstrated the activity of purified enzyme and crude extracts against *S. pneumoniae* strains including clinical isolates and different serotypes (Stoffels et al., 2017). Furthermore, we established the cultivation of the strain in a 100 L hanging bag photobioreactor system (Taunt et al., 2017; and unpublished data).

Although *C. reinhardtii* offers potential as a low-cost and benign expression host for products such as endolysins, to date, there have been few investigations into the recovery of recombinant proteins from microalgal hosts with the aim of identifying process challenges for scale-up. The choice of host cell can have significant implications on downstream processing (DSP). While the cell wall-reduced background does not represent a disadvantage for the cultivation at laboratory and pilot-scale [(Zedler et al., 2016), Stoffels et al. (unpublished data)], it remains to be investigated if such strains are suitable for large-scale cultivation and downstream processing.

Ultra Scale-Down (USD) technologies enable small scale experiments to be used in order to try and predict large scale bioprocessing results with greater ease and reduced time (Rayat et al., 2016). For instance, using a rotating shear device (RSD) (Boychyn et al., 2004), the shear experienced by cells in the feed zone of a centrifuge can be mimicked. This approach has been applied in previous studies to characterize the interaction between cell cultivation and process steps in product recovery (Lau et al., 2013; Voulgaris et al., 2016). By following shear device treatment with bench-top centrifugation the percentage recovery can be predicted. In a study with *E. coli*, it was shown that a weakening of cell strength during prolonged cultivation, resulted in an increase in product release rates from the cell and a decrease in clarification performance (Perez-Pardo et al., 2011).

This study examined the bioprocessing factors important for the harvest of a *C. reinhardtii* cell wall-reduced strain producing the Pal endolysin (strain TN72_pal). We applied the USD platform to further examine microalgal processing with the goal of establishing windows of operation for scale-up.

## Materials and Methods

### Cultivation of *Chlamydomonas reinhardtii* TN72_pal

Lab-scale cultures of *C. reinhardtii* TN72_pal were cultivated in Tris-acetate-phosphate (TAP) medium (Gorman and Levine, 1965) in illuminated incubators at 100 μmol.m^−2^.s^−1^, 120 rpm shaking and 25°C. At pilot-scale, TN72_pal was grown in an illuminated, temperature controlled room with a single-use hanging bag system provided by Supreme Biotechnologies Ltd (Nelson, New Zealand). Four cultivation bags of the system were filled with 25 L of autoclaved TAP medium each, sparged with air from the bottom and illuminated with 100 μE.m^−2^.s^−1^ using 10 Osram Lumilux Cool daylight fluorescence tubes. The cultures were cultivated for 3 days at 25°C and harvested at the end of the logarithmic phase [cell density at this stage: 0.5–0.6 g.L^−1^ dry cell weight (DCW)], when the yield of Pal in the culture is highest (Stoffels et al., 2017). The growth was monitored by optical density (750 nm) and DCW measurements.

### Pilot-Scale Continuous-Flow Intermittent-Discharge Disc-Stack Centrifugation

All disc-stack centrifugation experiments were performed with 100 L TN72_pal cultures. Cell harvest was carried out using either a GEA Westfalia CSA-1 at 9800 rpm and flow rates of 150 L.h^−1^ (Q/**Σ** 65 × 10^−9^ m.s^−1^), 200 L.h^−1^ (Q/**Σ** 87 × 10^−9^ m.s^−1^), and 250 L.h^−1^ (Q/**Σ** 109 × 10^−9^ m.s^−1^), or a GEA Westfalia Pathfinder PSC 1-06-177 (PSC-1) at 13,500 rpm (20,000 × g) with a flow rate of 120 L.h^−1^ (Q/**Σ** 95 × 10^−9^ m.s^−1^). The CSA-1 has 45 discs and a bowl volume of ≈0.6L and a solids holding space of ≈0.3L. The orifice height of the discharge mechanism of the CSA-1 is 4 mm and there are 6 slots in the outside of the bowl of ~41 mm width each. PSC-1 has 8 discs and a bowl volume of ≈1L and a solids holding space of ≈0.85L. Samples were taken before, during and after the centrifugation runs to determine the biomass recovery (analyzed by DCW), cell integrity (analyzed by particle size measurements) and the recovery of the recombinant protein Pal (determined by quantitative western blot analysis). To analyse the state of the cells and the product after entry in the centrifuge and before discharge, the bowl of the CSA-1 was opened during one run before the discharge of the cells as shown in Supporting Figure 1. Subsequently, the cells from the bowl were analyzed as described above.

### Ultra Scale-Down (USD) Centrifugation Studies

The USD centrifugation device has been described previously (Boychyn et al., 2004; Hutchinson et al., 2006; Tait et al., 2009; Li et al., 2013). Briefly, 20 mL samples were exposed to shear stress using a rotating shear device (RSD) consisting of a stainless steel chamber of 50 mm diameter and a height of 10 mm with a rotating disc of 40 mm diameter and 1 mm thickness (Tait et al., 2009). The RSD was operated at 6,000 and 12,000 rpm for 20 s, corresponding to shear at 4.5 × 10^4^ and 5.3 × 10^5^ W.kg^−1^, respectively. These were chosen to represent the typical shear experienced in the feed zone of hydro-hermetic and non-hermetic disc-stack centrifuges, respectively (Chatel et al., 2014). The sedimentation properties of sheared and non-sheared samples were characterized using a bench-scale centrifuge and Sigma theory (Ambler, 1959) in terms of equivalent settling area, Σ_*USD*_ in Equation 1 (Li et al., 2013):

(1) $$\begin{array}{r} \Sigma_{USD}=\;\frac{V_{USD}\omega^{2}}{2gln\left(\frac{2R_{O}}{R_{O}+R_{i}}\right)} \end{array}$$

where V_USD_ is the volume of process material in the centrifuge tube, ω is the radial speed, g is the acceleration due to gravity, R_i_ is the inner radius (the distance between the center of rotation and the top of the liquid), and R_o_ is the outer radius (the distance between the center of rotation and the bottom of the tube).

The suspension volume, *V*_*USD*_, and the spin time, *t*_*USD*_, represent the equivalent flow rate such that we have the following expressions, where *S* represents solids remaining in supernatant in Equation 2 (Rayat et al., 2016):

(2) $$\begin{array}{r} S=f\left(\frac{V_{USD}}{t_{USD}\Sigma_{USD}}\right)=f\left(\frac{Q_{FS}}{\Sigma_{FS}}\right) \end{array}$$

Following shearing, centrifugation was carried out in an Eppendorf 5424R benchtop centrifuge (Cambridge, UK) with a FA-45-24-11 (24 × 2 mL Eppendorf tubes) rotor at room temperature using 2 mL Eppendorf tubes and a 2 mL fill volume. For shake flask cultures, samples were centrifuged at 8,000 rpm for 5.5, 2.8, 1.8, 1.4, and 1.1 min giving V_USD_/(t_USD_*Σ*_USD_) values of 22 × 10^−9^ m.s^−1^, 43 × 10^−9^ m.s^−1^, 65 × 10^−9^ m.s^−1^, 87 × 10^−9^ m.s^−1^ and 109 × 10^−9^ m.s^−1^, respectively. For bag cultures, samples were spun at 8,000 rpm for 6 min, 6,200 rpm for 5 min and 5,600 rpm for 3 min giving V_USD_/(t_USD_*Σ*_USD_) of 22 × 10^−9^ m.s^−1^, 43 × 10^−9^ m.s^−1^ and 87 × 10^−9^ m.s^−1^, respectively. The top 1.5 mL of supernatant was carefully removed from each tube and used for analysis (Tait et al., 2009).

### Clarification Efficiency

The solids content in the supernatants after centrifugation was estimated by optical density measurements at 750 nm. The solids remaining, S was calculated (%) by Equation 3:

(3) $$\begin{array}{r} S=\;\frac{OD_{S}-OD_{O}\;}{OD_{F}-OD_{O}} \end{array}$$

Where OD_S_ is the optical density of the supernatant after centrifugation, OD_O_ is the optical density of a well-clarified supernatant (21,000 × g, 30 min, V_USD_/(t_USD_*Σ*_USD_) 4 × 10^−9^ m.s^−1^), and OD_F_ is the optical density of the feed (Li et al., 2013).

### Measurement of the Recombinant Protein Pal and Endogenous Proteins

The relative amount of the product Pal in each sample was determined by quantitative western blot analysis with anti-HA antibodies and IRDye^®^ secondary antibodies as described in Stoffels et al. (2017). The relative amount of the endogenous proteins D1 and RbcL was determined with anti-D1 or anti-RbcL antibodies and IRDye^®^ secondary antibodies, the total protein by Coomassie-stained SDS-PAGE. The Odyssey^®^ CLx Infrared Imaging System (LI-COR Biosciences) was used for quantification in all cases. The loss of Pal (%) to the supernatant was determined by western blot analysis of the supernatant and feed. The values of the feed were used as 100 %. To analyse the recovery of Pal (%) during disc-stack centrifugation, the measured amount of Pal in the discharged slurry or the cell paste from the bowl was normalized against the biomass content of the sample. Subsequently, the values could be compared to the amount of Pal per g of biomass in the culture before harvest, which was seen as 100%. The biomass content of the algal slurry or paste was determined by lyophilizing 5–20 ml samples in a freeze-dryer for 24–72 h and subtracting the dry weight of the salts in the same volume of TAP medium. To measure the biomass content of the culture before harvest, 50 ml of culture were pelleted by centrifugation and the pellets lyophilized for 24–72 h.

### Particle Size Distribution

Particle size distribution was measured using laser light diffraction (Mastersizer 3000, Malvern Instruments Ltd, Malvern, UK); Refractive index (RI) 1.41, absorption 0.01. Samples were added to deionized water in the dispersion unit operating at 1,200–1,500 rpm until the laser obscuration value was above 5%. Each sample was analyzed five times and the mean size distribution determined. In addition, the cells/particles were analyzed by light microscopy with a Zeiss Axio Lab A1 microscope and an AxioCam ERc5s camera.

## Results

### Prediction of Centrifugation Performance: USD for Solids Remaining and Product Loss to Supernatant

**Figure 1:**
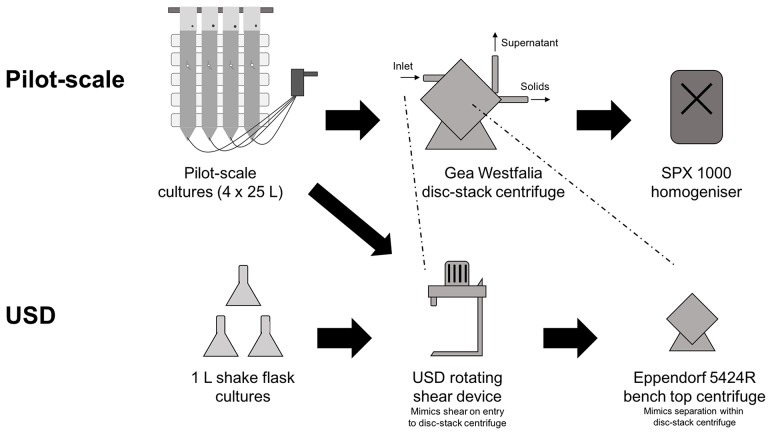
Flow sheet outlining the processing steps involved in both pilot-scale and USD studies. Pilot-scale cultures of *Chlamydomonas reinhardtii* TN72_pal were grown in a 4 × 25 L modular hanging bag photobioreactor system before harvest with Gea Westfalia continuous-flow, intermittent-discharge disc-stack centrifuges. Disruption of the cellular component at pilot-scale was performed using a SPX 1000 homogenizer. In the USD study, cultures were grown in 1 L shake flasks or the hanging bag system and then processed using the USD rotating shear device to mimic the shear cells are subject to upon entrance into the Gea Westfalia disc-stack centrifuge. Subsequently, a bench top centrifuge was used to mimic the separation of cells from culture media that occurs within the pilot-scale centrifuge.

The rotating shear device (RSD) is an established method that can be used to simulate the shear conditions in the feed zone of disc-stack centrifuges. In combination with a bench-top centrifuge, it has been used to predict the clarification efficiency of mammalian cell cultures and bacterial cultures in a pilot-scale disc-stack centrifuge (Rayat et al., 2016). To investigate whether this method can also be applied to predict the clarification efficiency of algal cultures, we exposed *Chlamydomonas reinhardtii* TN72_pal cells to low shear (4.5 × 10^4^ W.kg^−1^) and high shear (5.3 × 10^5^ W.kg^−1^) in the RSD, followed by bench-top centrifugation with a range of (V_USD_/(t_USD_*Σ*_USD_)) values. The comparison of pilot scale and USD flowsheets is outlined in Figure 1. Since this USD method can be performed with milliliters of culture, shake flask cultures can provide a sufficient volume for predictive studies. However, the type of mixing in shake flask cultures differs from the pilot-scale hanging-bag system, which likely results in differences in mass transfer, illumination and aeration. To analyse if this has an influence on the properties of the cells and their behavior during harvest, we performed the experiment with both types of culture. Subsequently, we analyzed the solids remaining in the supernatant (%) by optical density measurements and the release of the product Pal by western blot analysis.

**Figure 2:**
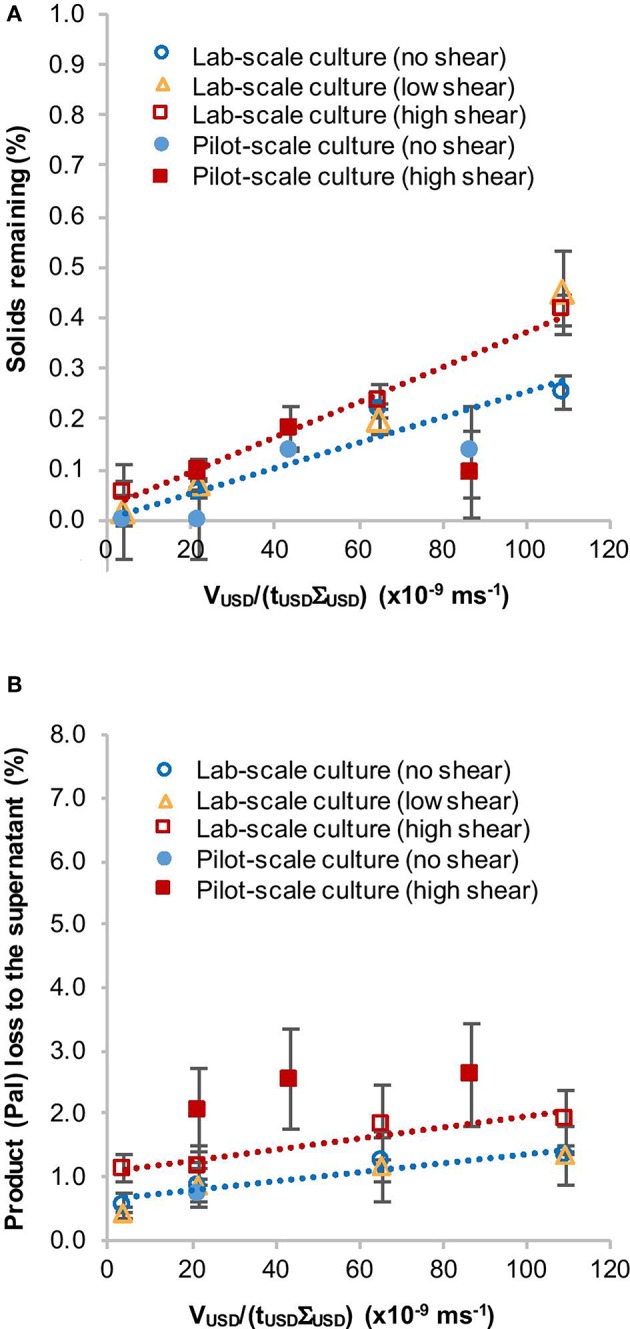
Comparison of the solids remaining (%) **(A)** and product loss (%) to the supernatant **(B)** after ultra scale-down (USD) centrifugation of a lab-scale and a pilot-scale *Chlamydomonas reinhardtii* TN72_pal culture. Both types of culture were exposed to two levels of shear in the rotating shear device followed by bench-scale centrifugation at equivalent settling area. Solids remaining (%) were determined by optical density measurements and the loss of the product Pal (%) to the supernatant by quantitative western blot analysis. The same pilot-scale culture was used for the 200 L.h^−1^ GEA Westfalia CSA-1 run (see Figure 3 and Table 1). The linear regression lines correspond to the lab-scale culture data for high shear (red) and no shear (blue). Error bars represent ± 1 SD (*n* ≥ 3).

Overall the solids remaining in the supernatant were below 0.5% for both low shear and high shear conditions and the amount of product found in the supernatant ranged from 0.4 to 2.6% (Figure 2, Table 1). Clarification efficiency decreased with increasing V_USD_/(t_USD_*Σ*_USD_) values and loss of Pal to the supernatant was greater for samples that had been exposed to high shear in the RSD. This indicates an influence of the RSD treatment on the cell integrity. Furthermore, we did not observe a difference between the shake flask and pilot-scale culture concerning the solids remaining (%) in the supernatant (Figure 2A, Table 1). However, the average amount of Pal lost to the supernatant was slightly higher in the experiment with the pilot-scale culture (Figure 2B). This is more likely due to general variations between individual cultures and western blot analyses, which can be seen by the overlapping standard deviations in Figure 2B and the range of standard deviations in Table 1. Taken together, this suggests that a shake flask-grown *C. reinhardtii* TN72 culture can be used to predict the behavior of a pilot-scale culture in this USD method.

**Table 1:** Comparison of solids remaining (%) and product loss (%) between ultra-scale-down (USD) experiments and continuous-flow, intermittent-discharge disc-stack centrifugation.

**Centrifuge/equipment**	***Chlamydomonas reinhardtii* TN72 culture**	**V_USD_/(t_USD_Σ_USD_) or Q/Σ (x10^−9^ ms^−1^) (Equiv. flow rate CSA1)**	**Solids remaining (%)**			**Product (Pal) loss to the supernatant (%)**		
			**No shear**	**Low shear**	**High shear**	**No shear**	**Low shear**	**High shear**
**USD experiments** (Rotating Shear Device (RSD) and benchtop centrifuge)	Lab-scale culture (Shake flask culture at late log phase)	4.0 (Well spun)	0.00± 0.00	0.02± 0.03	0.05± 0.05	0.58± 0.16	0.43± 0.09	1.14± 0.21
		21.7 (50 L/h)	0.05± 0.00	0.07± 0.03	0.09± 0.03	0.87± 0.37	0.87± 0.26	1.17± 0.30
		65.1 (150 L/h)	0.22± 0.00	0.20± 0.03	0.23± 0.03	1.28± 0.34	1.16± 0.53	1.86± 0.60
		109.0 (250 L/h)	0.25± 0.03	0.45± 0.08	0.41± 0.03	1.34± 0.46	1.38± 0.03	1.93± 0.44
	Pilot-scale culture (100 L) 2	21.7 (50 L/h)	0.00± 0.08	NA	0.10± 0.02	0.73± 0.23	NA	2.07± 0.66
	(late log phase)	43.4 (100 L/h)	0.14± 0.00	NA	0.18± 0.04	NA	NA	2.54± 0.80
		86.8 (200 L/h)	0.14± 0.09	NA	0.09± 0.09	NA	NA	2.62± 0.83
**GEA Westfalia CSA-1**	Pilot-scale culture (100 L) 1 (late log phase)	65.1 (150 L/h)	0.24 ± 0.06 (Range 0.20–0.35)			2.76 ± 0.63 (Range 2.23–3.64) Well spun: 1.65 ± 0.32		
	Pilot-scale culture (100 L) 2 (late log phase)	86.8 (200 L/h)	0.40 ± 0.14 (Range 0.14–0.57)			1.63 ± 0.43 (Range 0.98–2.08)		
	Pilot-scale culture (100 L) 3 (late log phase)	109.0 (250 L/h)	0.18 ± 0.04 (Range 0.13–0.23)			1.67 ± 0.81 (Range 0.75–2.57)		
	Pilot-scale culture (100 L) 4 (stationary phase)	43.4 (100 L/h) 86.8 (200 L/h)	0.51 ± 0.05 2.10 ± 0.19			NA		
**GEA Westfalia Pathfinder PSC-1**	Pilot-scale culture (100 L) 5 (mid log phase)	110–120 L/h	1.26 ± 0.20 (Range 1.12–1.55)			6.69 ± 2.29 (Range 5.00–10.42)		

*Low shear: 4.5 × 10^4^ W kg^−1^; High shear: 5.3 × 10^5^ W kg^−1^. ± 1 SD (n ≥ 3) is shown in the table*.

### Pilot-Scale Centrifugation Using a GEA Westfalia CSA-1 and Comparison to USD Data

**Figure 3:**
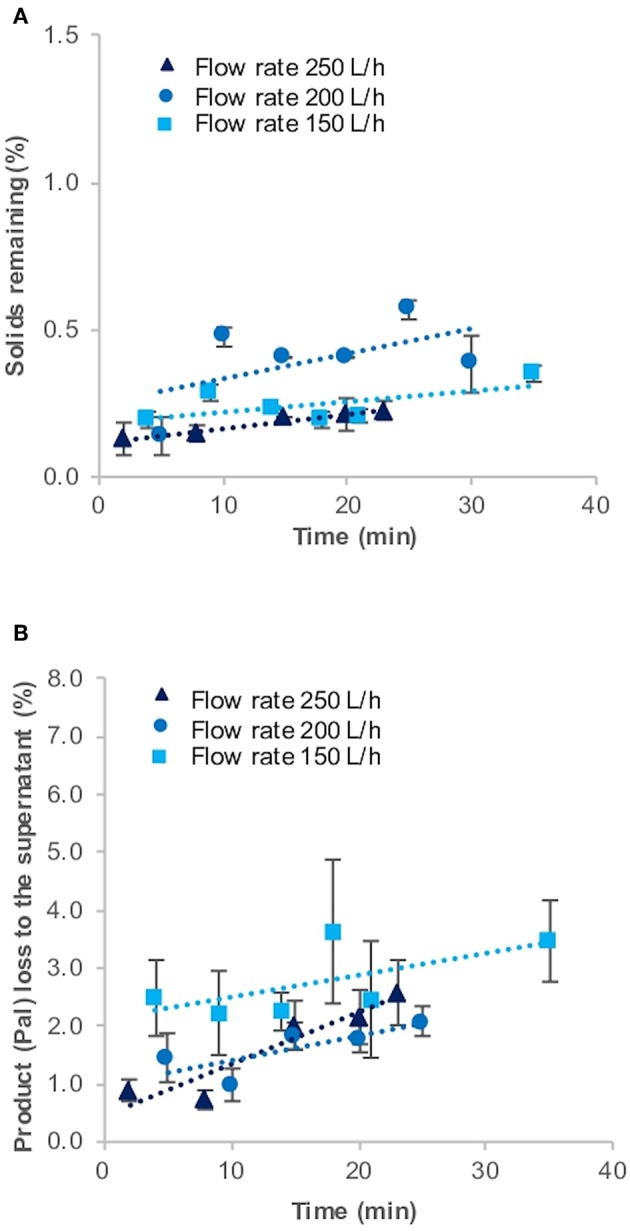
Solids remaining (%) **(A)** and product loss (%) **(B)** to the supernatant during harvest of *Chlamydomonas reinhardtii* TN72_pal by continuous-flow disc-stack centrifugation. Three 100 L pilot-scale cultures were harvested in a GEA Westfalia CSA-1 at flow rates of 150 L.h^−1^ (Q/**Σ** 65 × 10^−9^ m.s^−1^), 200 L.h^−1^ (Q/**Σ** 87 × 10^−9^ m.s^−1^), and 250 L.h^−1^ [(Q/**Σ** 109 × 10^−9^ m.s^−1^], respectively. Solids remaining (%) in the supernatant was determined by optical density measurements and the loss of the product Pal (%) to the supernatant by quantitative western blot analysis. Error bars represent ± 1 SD (*n* ≥ 3).

To test whether this USD method can be used to predict the harvest in a pilot-scale disc-stack centrifuge, we performed three harvests of 100 L *C. reinhardtii* pilot-scale cultures (harvested at late log phase) using the CSA-1 disc-stack centrifuge at different flow rates. During all three CSA-1 runs, the solids remaining (%) were in the range of 0.13–0.57% with an average of 0.27% ± 0.11. In comparison, the USD experiments performed at high shear and bench-top centrifugation with V_USD_/(t_USD_Σ_USD_) values equivalent to flow rates of 150–250 L.h−1, resulted in solids remaining (%) values of 0.09–0.41% with an average of 0.24% ± 0.16. The amount of Pal in the supernatant during all CSA-1 runs stayed between 0.75 and 3.64% with an average of 2.0% ± 0.6 (Figure 3, Table 1), whereas the USD experiments resulted in a range of 1.9–2.6% with an average of 2.1% ± 0.4 (Table 1). These results show a close correlation between the USD experiments and the comparable pilot-scale runs, and suggest that this USD method can be used to predict the clarification efficiency and product loss for *C. reinhardtii* TN72 cultures in the CSA-1 disc-stack centrifuge.

However, it was not possible to see a trend depending on the different flow rates during the three CSA-1 runs. In general, the solids remaining (%) are expected to increase when the flow rate is increased. Because of the high clarification efficiency, we used the three highest possible flow rates on the CSA-1, which do not represent a wide spread of Q/sigma values. Furthermore, all three runs were performed with independent 100 L pilot-scale cultures. The results presented in Figure 3 suggest that culture-to-culture variations are more pronounced and can mask differences caused by small changes in the flow rate (Q/sigma values). We also performed a CSA-1 harvest with a stationary phase culture, which resulted in a nearly eight times higher solids remaining (%) value at 200 L.h^−1^ (2.10 ± 0.19), most likely caused by the declining health of the cells (Table 1). In addition, we observed a slight increase of solids remaining (%) and product loss (%) over time during all runs. This can be explained by the increasing amount of cells in the centrifuge bowl and the lysis of some cells over time.

### Influence of the Discharge on the Integrity of Cells and Product

**Figure 4:**
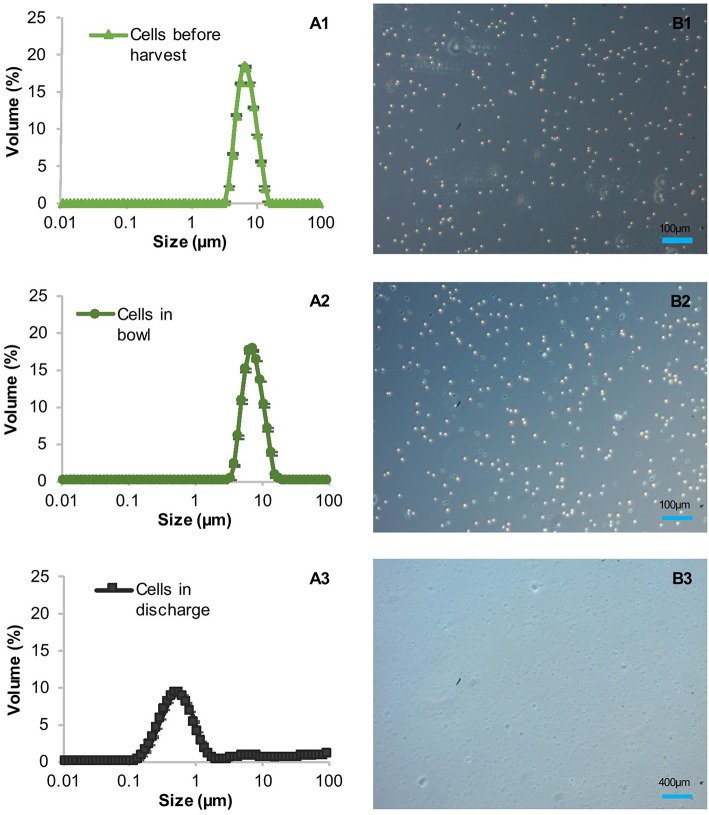
Integrity of *Chlamydomonas reinhardtii* TN72_pal cells during harvest by continuous-flow intermittent-discharge disc-stack centrifugation. A 100 L pilot-scale culture was harvested in a GEA Westfalia CSA-1 at a flow rate of 150 L.h^−1^ (Q/Σ 65 × 10^−9^ m.s^−1^). Samples were taken before harvest, from the centrifuge bowl during the run and the discharge after the harvest. The cell integrity was determined by particle size measurements **(A1–A3**) and light microscopy **(B1–B3)**. Error bars represent ± 1 SD (*n* ≥ 5).

**Figure 5:**
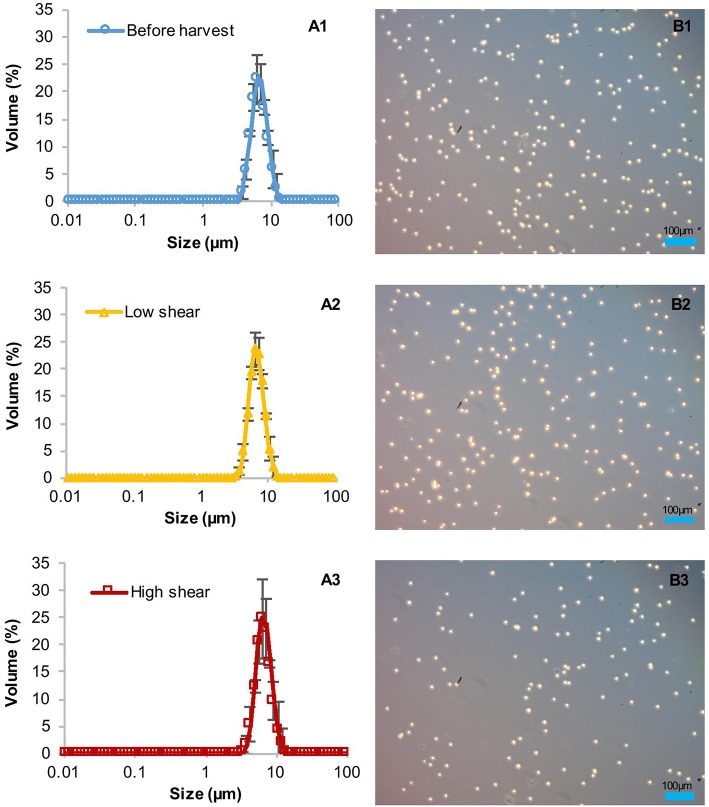
Integrity of *Chlamydomonas reinhardtii* TN72_pal cells before and after ultra scale-down (USD) centrifugation. For the USD centrifugation studies, cells from two pilot-scale bag cultures were exposed to two levels of shear in the rotating shear device followed by bench-scale centrifugation at equivalent settling area. The cell integrity was determined by particle size measurements **(A1–A3)** and light microscopy **(B1–B3)**. Error bars represent ± 1 SD (n≥5).

The high clarification efficiencies and low amounts of product in the supernatant suggested initially that the shear forces in the CSA-1 do not harm the cell wall-reduced TN72 cells, at least not during entry into the centrifuge. However, particle size measurements indicated that the cells were damaged before the subsequent homogenisation step (Figure 4). To ascertain the cause of cell damage, we stopped the centrifugation in the middle of one CSA-1 run and opened the bowl to analyse the cells after entry into the centrifuge but before discharge as shown in Supporting Figure 2. The particle size distribution of the cells was measured with the Mastersizer 3000 and cell integrity was assessed using light microscopy. Both methods showed intact cells after entry into the bowl, but revealed a strong reduction in particle size after discharge (Figure 4). This strongly suggests that cells break into cell debris during discharge from the CSA-1. In contrast, cells treated by the above described USD method were still intact after the treatment and comparable to cells in the bowl or before harvest (Figure 5). Taken together, these results suggest that the RSD can be used to predict the shear forces in the feed zone of the CSA-1, which have an influence on the clarification efficiency and the amount of product lost to the supernatant. However, the method cannot predict the forces TN72 cells experience during discharge from the CSA-1.

**Figure 6:**
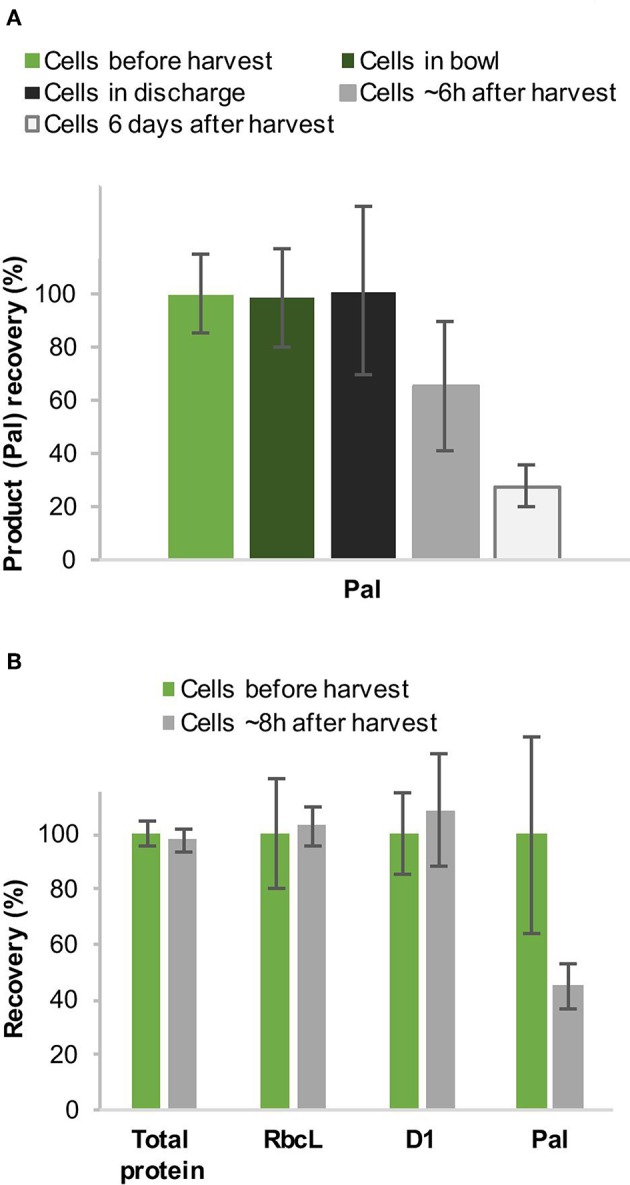
Recovery (%) of the recombinant protein Pal during harvest by continuous-flow intermittent-discharge disc-stack centrifugation **(A)** and comparison to the recovery of endogenous proteins **(B)**. *Chlamydomonas reinhardtii* TN72_pal pilot-scale cultures were harvested in a GEA Westfalia CSA-1 at a flow rate of 150 L.h^−1^ (Q/Σ 65 × 10^−9^ m.s^−1^) **(A)** and 250 L.h^−1^ [(Q/Σ 109 × 10^−9^ m.s^−1^] **(B)**, respectively. The amount of Pal, D1 and RbcL in the cell material was determined by quantitative western blot analysis, and the total protein by Coomassie-stained SDS-PAGE. Error bars represent ± 1 SD (*n* ≥ 3).

In addition, we observed a strong reduction of the product in the cell material when we analyzed the amount of Pal a few hours after disc-stack centrifugation before the subsequent homogenization step. Since there were only low amounts of Pal in the supernatant during disc-stack centrifugation (1.6–2.8%, see Figure 3B, Table 1), we investigated this further to close the mass balance. We performed western blot analysis with cells before harvest as well as from the bowl and discharge. Two sets of samples were prepared, the first were frozen in liquid nitrogen immediately after sampling and the second set was left for several hours/days on ice to simulate long holding or processing times at large-scale. The western blot analysis indicated a strong reduction of Pal within the first 6–8 h and a further, but slower, reduction within the following days (Figure 6). In contrast, an overall protein analysis by Coomassie-stained SDS-PAGE and western blot analyses with antibodies specific to the endogenous proteins RbcL (RuBisCO large chain) and D1 (subunit of photosystem II) did not show the same reduction within 8 h, which suggests a higher sensitivity of Pal to degradation (Figure 6B).

### Comparison to GEA Westfalia Pathfinder PSC-1 and Homogenization

**Figure 7:**
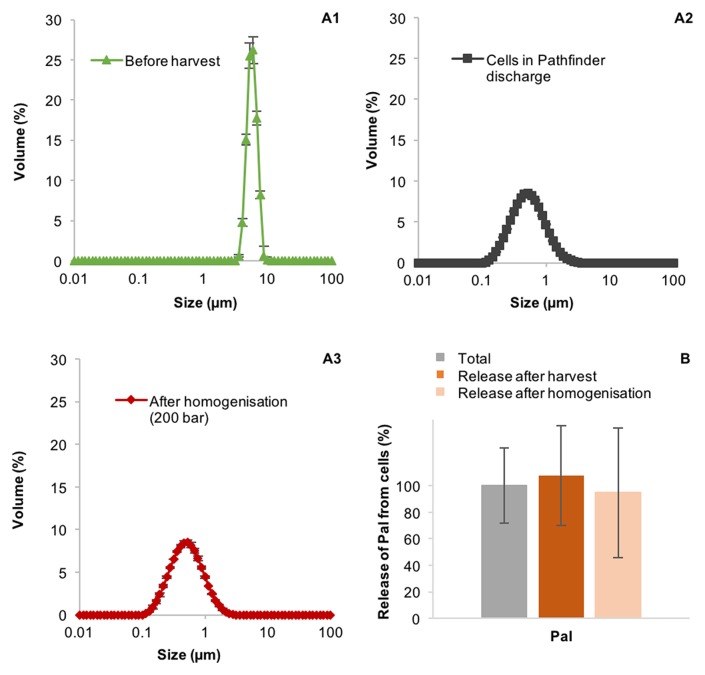
Integrity of *Chlamydomonas reinhardtii* TN72 cells **(A1–3)** and product release **(B)** during harvest with a GEA Westfalia Pathfinder PSC-1 and subsequent homogenization. A 100 L pilot-scale culture was harvested at a flow rate of 120 L·h^−1^ (Q/ 95 × 10^−9^ m·s^−1^). Subsequently, the cells were treated with a SPX 1000 homogenizer. The cell integrity was determined by particle size measurements and the amount of the product Pal by western blot analyses. Error bars represent ± 1 SD (*n* ≥ 4).

The CSA-1 disc-stack centrifuge has a relatively slow discharge mechanism. It takes ~0.5 s for the bowl to fully open and it stays open for 10 s. This might result in periods where the solids are discharging through a smaller orifice, which might cause greater shear. To analyse if the breakage of the TN72 cells during discharge is specific to the CSA-1 mechanism, we performed one harvest with the, newer GEA Westfalia Pathfinder PSC-1. This centrifuge has a hydrostop system, which allows the bowl to open in <0.1 s and the time the bowl is open during discharge can be set to as little as 0.1 s. The particle size distribution of the cells before and after centrifugation was analyzed with the Mastersizer 3000 and light microscopy. However, both methods showed again a strong reduction in particle size after centrifugation (Figure 7), and shown in Supporting Figure 3, indicating that the discharge mechanism of the PSC-1 also results in cell breakage of TN72. Furthermore, subsequent homogenization of the harvested cells at 200 bar using a SPX 1000 homogenizer (APV), had little impact on the particle size distribution or release of Pal (Figure 7).

## Discussion

### Scale-Up of Recombinant Protein Production in *Chlamydomonas reinhardtii*

In the last few years over 100 recombinant proteins have been made in the chloroplast of the green alga *Chlamydomonas reinhardtii* (Dyo and Purton, 2018), but only a few studies went on to investigate the scale-up of cultivation. Gimpel et al. (2015) investigated the greenhouse cultivation of *C. reinhardtii* (with a wild type cell wall) in a 100 L bag system and achieved biomass yields of 0.3 g.L^−1^ (DCW) with up to 3.3 mg.L^−1^ of bovine Milk Amyloid A protein. The pilot-scale cultivation of TN72 has been previously performed for the production of a cytochrome P450 enzyme (CYP79A1) and a diterpene synthase (TPS4) in a 100 L glass Green Wall PBR by Zedler et al. (2016). In this study, both TN72 strains also reached biomass yields of 0.3 g.L^−1^ (DCW), which has shown that the cell wall-reduced strain performs comparably to the wild type during pilot-scale cultivation and is robust enough to enable outdoor cultivation in Green Wall PBRs. The expressed proteins accumulated in the PBRs to levels of 6.77 mg.L^−1^ (CYP79A1) and 22 mg.L^−1^ (TPS4). However, the study presented here is the first to consider the downstream bioprocessing challenges unique to using a cell wall-reduced microalga such as TN72 for protein production.

### Performance of USD and Scale-Up of Centrifugation

USD centrifugation is a technique for studying scale-up of cell harvest. In accordance with previous work on other expression systems such as *E. coli* and Chinese Hamster Ovary cells, the ability of USD to predict clarification was examined (Rayat et al., 2016). *E. coli* USD studies have obtained similar values for the percentage of solids remaining in the supernatant (Chatel et al., 2014), reaffirming that the rotating shear device is able to predict pilot-scale centrifuge clarification performance.

A comparison of *C. reinhardtii* TN72_pal to a previous USD study with *E. coli* (Chatel et al., 2014), with both operating at 100 L/h equivalent, gives ~0.15 and 8% solids remaining, respectively. In a separate study, CHO cells were found to have a clarification efficiency with 3–5% solids remaining for 100 L.h^−1^ equivalent flow rate through the centrifuge (Hogwood et al., 2013), although no percentage clarification comparison with scale-up data was recorded. Of the host organisms, the order (best–worst) of % clarification is *C. reinhardtii* TN72 > CHO cells > *E. coli*. By their very nature, CHO cells are shear sensitive since animal cells lack a cell wall. When centrifuged, even under low shear conditions, cells are more likely to rupture and release intracellular contents into the culture media–resulting in poorer clarification post-centrifugation (Hogwood et al., 2013). *E. coli* and *C. reinhardtii* cells possess cell walls and are therefore more resilient to shear stresses. The cell wall of wild type *C. reinhardtii* is a multi-layered structure composed mainly of hydroxyproline-rich glycoproteins (Voigt and Frank, 2003), whereas the TN72 strain carries the cw15 mutation which results in cells with only a residual cell wall (Davies and Plaskitt, 1971). However, it is likely that this minimal wall provides increased resiliency when compared to a true protoplast or to mammalian cells.

*E. coli* cultures can be grown to higher cell densities [13 g.L^−1^ DCW (above study)] than *C. reinhardtii* [0.5–0.6 g.L^−1^ DCW (this study)], which results in a higher viscosity of the culture. Furthermore, *E. coli* cells are significantly smaller than *C. reinhardtii* cells (a volume of ~0.7 μm^3^ compared to ~140 μm^3^). Both factors mean that *E. coli* cultures have slower sedimentation rates during harvest by centrifugation, which results in lower clarification efficiency. Furthermore, both *E. coli* and *C. reinhardtii* have motile cells with flagella, which reduces the sedimentation rate to a higher degree in denser cultures.

### Limitations of USD Predictions

In the case of Pal, this USD method overestimates the yield because it cannot account for cell damage on discharge, where the product is more likely to be readily exposed to degradation. The ultra scale-down (USD) technique mimicking centrifugation is based on the principle that significant hydrodynamic stress will be encountered by the process material in the feed zone of the centrifuge before it enters the settling region (Chatel et al., 2014). In the case of TN72, cells are sufficiently resilient to remain intact through the feed zone, as shown by the bowl sampling experiments (Figure 5) and Supporting Figures 2, 3.

Previous work on understanding the impact of shear forces in cell discharge for the periplasmic expression of an antibody fragment (Fab) in *E. coli* showed that cell breakage increases with discharge velocity (Aucamp et al., 2014). In this work, an attempt was made to create a capillary device for USD prediction of cell damage due to discharge. While this could rank strains on their susceptibility to shear damage, on average two to three times more Fab was released from the pilot-scale discharge than with the USD mimic.

In order to contrast the product loss between different types of centrifuge, the recovery of Pal was examined in the PSC-1 centrifuge. The discharge mechanism was significantly improved by GEA Westfalia between the CSA-1 (c 1990's) and PSC-1 (c 2010's). Improvement to the control of the hydraulics by a hydrostop system that moves the sliding piston in the PSC-1 means that the mechanism opens and closes in <0.1 s. This minimizes the time the discharged material is pushed through an orifice smaller than the fully open hole, which was expected to decrease the shear. Nonetheless, cells are sufficiently weakened and are still broken upon exit from the PSC-1.

### Implications of Cell Breakage During Disc-Stack Centrifugation

It can be observed that there is a significant loss of Pal from the extract within a few hours after centrifugation as measured by western blot analysis (Figure 6). We hypothesize that the loss of Pal is most likely caused by proteolytic activity in the damaged cells. The endolysin Pal is naturally produced and accumulates in *Streptococcus* cells and is therefore adapted to resist prokaryotic proteases. The chloroplast, which originated from a cyanobacterium, has mainly prokaryotic-type proteases (Nishimura et al., 2016) and Pal shows a high stability in the chloroplast of unbroken cells (Stoffels et al., 2017). However, after cell breakage eukaryotic proteases from the cytoplasm can access Pal, which is a likely explanation for the observed reduction of Pal. This appears to be specific to the recombinant protein, as levels of two indicative host cell proteins, RbcL and D1 remain relatively constant throughout processing. This indicates the degradation during extended processing time may be unique to Pal, and other recombinant proteins may be more resilient. It is difficult to avoid long processing times during large-scale production processes. Therefore, it is important that a biological product is stable in harvested or broken cells of the production host for several hours. For the recovery of intracellular products that are more resistant to degradation, cell breakage of TN72 during harvest could be used as an advantage, since this feature can help to decrease the number of DSP steps. Significant capital and operational costs could be saved, if a homogenisation step is not required for the release of an intracellular product. Furthermore, it could reduce the overall processing time, especially since the throughput of homogenizers is limited and many cell types (e.g. *E. coli)* require several homogenisation cycles for full breakage (Li et al., 2013). A reduction of the total processing time can save costs and reduce the risk of degradation of the product.

### Future Work

As *C. reinhardtii* strains such as TN72 represent a promising cell platform for production of various recombinants including endolysins, subunit vaccines and bioactive compounds (Zedler et al., 2015; Taunt et al., 2017; Charoonnart et al., 2018), an understanding of the bioprocessing of this host is important. Optimisation of biomass and recombinant protein yields via strategies such as fed-batch cultivation (Fields et al., 2018) will be key to improving process economics. Linking upstream and downstream processing, for instance by investigating nutrient optimisation, and the influence of cultivation conditions on the biomass productivity as well as the bioprocessing characteristics of TN72 should be investigated. In order to recover intact cells for whole cell therapeutics such as oral vaccines (Rosales-Mendoza et al., 2015), alternatives to cell harvest will be required. This may take the form of amendments to the centrifuge or batch processing without discharge. A tubular bowl centrifuge could be used as an alternative, since the discharge mechanism causes significantly less shear. However, the shear forces on entry are greater compared to disc-stack centrifuges and it would impact throughput both in terms of flow-rate that can be achieved, and time for deceleration prior to desludge. Alternatively, the use of Tangential Flow Filtration (TFF) or other membrane technologies for the harvest of whole-cell products could to be explored. Furthermore, *C. reinhardtii* wild type strains with a full multi-layered cell wall, which require harsher treatments for transformation and cell breakage at lab-scale (Economou et al., 2014), are likely more resilient to the discharge mechanism of disc-stack centrifuges and their bioprocessing characteristics should be investigated for whole cell products. Future work should examine the influence of time on processing, in particular if long holding periods cause weakening of the cell wall. Strategies to reduce the degradation of sensitive products such as Pal, could be the addition of enzymatic or chemical protease inhibitors. Longer term, TN72 could be further modified as an expression host to facilitate protein accumulation and downstream processing, for instance by the creation of protease knock-out strains using emerging CRISPR-cas technologies (Ferenczi et al., 2017). Recombinant protein production in conventional expression systems is today primarily done in protease knock-out strains such as *E. coli* BL21(DE3) or *Bacillus subtilis* WB800 (Jia and Jeon, 2016; Liu et al., 2018), since product yields and stability are increased in these strains. This approach has great potential to improve protein stability in *C. reinhardtii* TN72 and optimize it as an expression system.

## Conclusion

In this study we have presented scale-up of microalgal DSP for recovery of cell wall-reduced cells expressing recombinant protein. The cell wall-reduced TN72 strain was used as a production system for the expression of Pal endolysin in the chloroplast. Pilot-scale cultivation of recombinant TN72 was conducted in a modular hanging bag system, and cell harvest was performed using disc-stack centrifuges. While the overall solids recovery was high, the shear sensitivity of TN72 was shown to cause cell breakage on the discharge from both CSA-1 and Pathfinder PSC-1 centrifuges. Ultra scale-down methodologies were applied to understand if they were reliable predictors of shear sensitivity and centrifugation. It was demonstrated that USD could predict clarification efficiency and product release due to shear forces at the centrifuge inlet. It was also shown that flask-scale cultivation and processing in USD could effectively predict pilot-scale data. However, the USD centrifugation could not predict the cell breakage on discharge. Furthermore, we observed product degradation in the damaged cells in the subsequent hours. For TN72 to be considered as an industrial production system, future work is needed on investigating product stability to improve yields. Furthermore, lower shear methods of harvest and/or *C. reinhardtii* strains with a wild type cell wall need to be investigated in particular to enable the recovery of intact cells for whole cell products. This study represents a first step toward understanding the challenges for scale-up of recombinant protein production in a microalgal host.

## Data Availability Statement

The datasets generated for this study are available on request to the corresponding author.

## Author Contributions

BP and SP conceived the study. LS, BP, and SP analyzed data, interpreted results, drafted the manuscript, and designed the lab experiments. LS conducted all of the experimental work, data collection and calculations. AF performed parts of the USD study including data collection and calculations. GM helped to design and supervise the pilot scale centrifugation studies. All authors read and approved the final manuscript.

### Conflict of Interest

The authors declare that the research was conducted in the absence of any commercial or financial relationships that could be construed as a potential conflict of interest.
