# Supplementary material for: Downstream Processing of Chlamydomonas reinhardtii TN72 for Recombinant Protein Recovery
Source: Front Bioeng Biotechnol. 2019 Dec 6;7:383. doi: 10.3389/fbioe.2019.00383 (PMC6908742; doi:10.3389/fbioe.2019.00383)
Supplement: Supplementary file 1 [file Data_Sheet_1.docx]

**Supporting figure 1:** Opening the bowl of the GEA Westfalia CSA-1 during a run to sample cells of *Chlamydomonas reinhardtii* TN72 after entry and before discharge from the centrifuge.


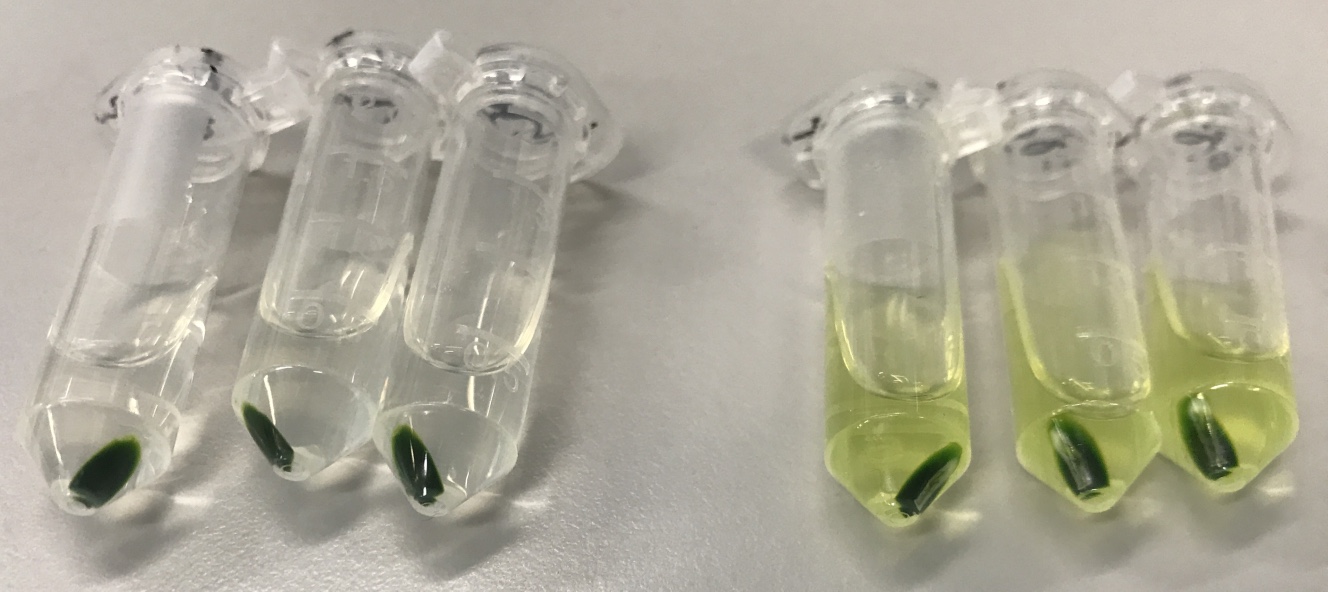


**Supporting figure 2:** Comparison of cells and culture supernatant during disc-stack centrifugation with a GEA Westfalia CSA-1. The culture supernatant of samples from the centrifuge bowl appear clear (left), whereas the culture supernatant of samples from the centrifuge discharge show a green color due to the release of chlorophyll (right). Furthermore, white pellets indicate the release of starch.

**Supporting figure 3:** Integrity of *Chlamydomonas reinhardtii* TN72 cells before (left) and after (right) harvest with a GEA Westfalia Pathfinder PSC-1. The cell integrity was determined by light microscopy.
